# Environmental protection via optimal global economic restructuring

**DOI:** 10.1016/j.mex.2019.03.017

**Published:** 2019-04-05

**Authors:** Alexander Vaninsky

**Affiliations:** City University of New York, Hostos Community College, United States

**Keywords:** Stochastic Data Envelopment Analysis with Perfect Object, Energy-environmental efficiency, Optimal economic restructuring, Stochastic data envelopment analysis, Systems of differential-algebraic equations, International trade and cooperation

## Abstract

This research proposes an approach to managing the environmental efficiency of the global economy via the optimal economic restructuring. The objective is to promote an increase in energy-environmental efficiency that supports economic growth constrained by greenhouse gas mitigation and energy conservation. The proposed method suggests the way of structural change in a global economy that the national economies should follow by economic restructuring, international cooperation, and trade. By following the recommended course of action, it becomes possible to increase the energy – environmental efficiency of the global economy as a whole via harmonized modifications in output per capita, use of renewable energy, and decrease in energy- and greenhouse gas emissions intensities. A novel stochastic data envelopment analysis with a perfect object method (SDEA PO) is utilized as a mathematical tool. A system of differential-algebraic equations is derived, leading to the determination of the trajectory of locally optimal structural change.

• This study introduces a novel method aimed to guide the optimal economic restructuring of the global economy via economic growth constrained by energy conservation and greenhouse gas mitigation.
• The suggested model provides guidance for energy-environmentally friendly economic restructuring of the global economy.

## Graphical abstract

**Figure:**
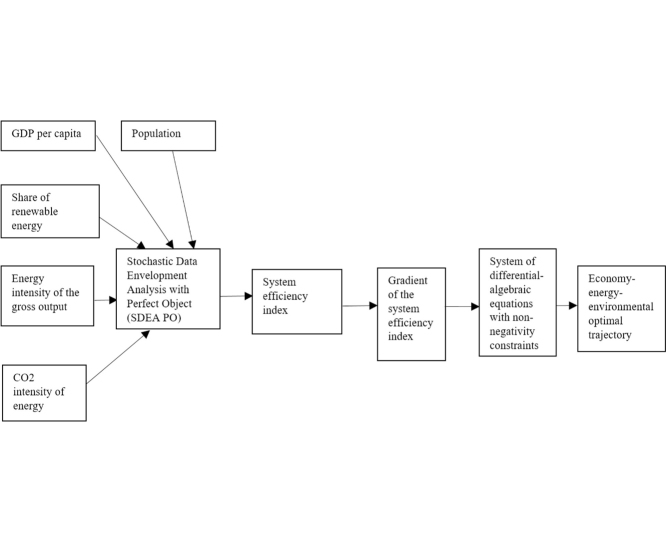

**Specifications Table**

**Table:** 

Subject Area:	*Energy*
More specific subject area:	*Optimal economic growth constrained by greenhouse gas mitigation and energy conservation*
Method name:	*Stochastic Data Envelopment Analysis with Perfect Object*
Name and reference of original method:	*A. Vaninsky, DEA with a Perfect Object: Analytical Solutions, Comm Mathematics and Applications, 2(1), (2011), 1–13.*http://www.rgnpublications.com/journals/index.php/cma/article/view/127/123.
	*A. Vaninsky, Stochastic DEA with a Perfect Object and Its Application to Analysis of Environmental Efficiency, Amer J Applied Mathematics and Statistics, 1(4), (2013), 57–63.*http://pubs.sciepub.com/ajams/1/4/2.
Resource availability:	*This study introduces a mathematical model*

## Method details

The 21st century presents an urgent need for a sustainable balance between economic development, environmental protection and energy consumption. These aims are ostensibly at odds. The continuing growth of the human population and rising standards of living are contingent on higher energy consumption. Predominantly, this leads to more pollution and waste from industrial, agriculture and construction activities. Environmentally-intelligent economic policy must be guided by principles of equity and sustainable development and complementary strategies of adaptation and mitigation, as, for example, the publication [1] has stated. This requires that national and regional economies pool resources and coordinate efforts, allowing the world economy to grow while environmental issues are simultaneously addressed. Climate action is, therefore, a collective problem on a global scale. Carbon dioxide (CO_2_) emissions are among the main factors of anthropogenic climate change. Although they can be pinpointed to specific geographical locations, emissions amass and disperse globally, impacting all countries and regions. Energy consumption is the main source of CO_2_ emissions, and this study specifically focuses on the mitigation of the emissions of this type. This problem turns out to be closely related to environmental protection and economic development that brings us to their simultaneous investigation as the factors of environmental efficiency.

In this paper, we develop a tool able to guide the restructuring of the global economy in a way that delivers the greatest possible contribution to environmental protection. To achieve this goal, we develop a mathematical model of energy-environmental efficiency. By using this model, we estimate the dynamics of the efficiency level as a function of structural change of the global economy. We also determine the structural change needed to increase it in the optimal way. To achieve this goal, we derive a system of differential-algebraic equations guiding the locally optimal restructuring of the global economy in time.

## Mathematical model: stochastic data envelopment analysis with a perfect object

A mathematical model presented in this study is based on the data envelopment analysis (DEA) developed in [2,3]. A conventional DEA considers a group of objects referred to as decision making units (DMUs) that use a vector of inputs ***X*** to produce a vector of outputs ***Y***. By means of a linear programming (LP) technique, DEA weighs inputs and outputs of each DMU against all other DMUs in the group and generates an efficiency index *E* scaled to the interval from 0 to 1. Based on the efficiency index, the DMUs are sorted into efficient (*E* = 1) and inefficient (*E* < 1) categories. Inefficient DMUs are expected to acquire the best practices of the efficient ones, thus improving their performance.

The DEA efficiency measure is based on the efficiency ratio suggested in [4]:

(1) $$E=\frac{\sum_{i=1}^{s}u_{i}Y_{i}}{\sum_{j=1}^{r}v_{j}X_{j}},$$

(2) $$0\leq E_{}\leq 1.$$

where ***u*** = (*u*_1_,…, *u_s_*) ≥ 0 and ***v*** = (*v*_1_,…, *v_r_*) ≥ 0 are the weight coefficients assigned to the outputs and inputs, respectively. DEA allows for the evaluation of the weight coefficients using a series of LP optimization problems, one for each DMU in the group. Details may be found, among other sources, on the website http://deazone.com/.

This research utilizes Stochastic DEA with a perfect object (SDEA PO) invented in [5,6]. Publication [5] suggested to append the group of actual DMUs with a perfect object (PO) having the smallest inputs and greatest outputs in the group. The perfect object is a virtual DMU serving as an example of a best practice and a benchmark for efficiency comparisons. The addition of the PO resulted in an explicit formula for the efficiency index that allowed to obviate the use of linear programming.

With a perfect object added, an explicit formula for the efficiency score of a DMU is as follows, [5]:

(3) $$E=\;\underset{0\leq i\leq s}{\max}\frac{Y_{i}}{Y_{\mathrm{0i}}}\underset{0\leq j\leq r}{\times \max}\frac{X_{\mathrm{0j}}}{X_{j}}=\left(\frac{Y_{i*}}{Y_{\mathrm{0i}*}}\right)\;\left(\frac{X_{\mathrm{0j}*}}{X_{j*}}\right)$$

where *Y*_i_ and *X_j_* are the *i*-th output and *j*-th input, respectively, i* and j* are the output and input corresponding to the maximum relative values, and lower index 0 stands for the perfect object. This version of the DEA was referred to as DEA with a perfect object (DEA PO) in [5]. As follows from the Formula (3), the DEA PO efficiency index is a product of maximum relative output and the inverse of the minimum relative input. Using the inverse value of the minimum relative input in the Formula (3) is more convenient for the stochastic version of DEA PO, since both terms are scaled to the interval [0,1].

Conventional DEA, in any form, is not fully suitable for global scale environmental studies. Since all objects exist in a shared environment, the inefficiency of one spreads to all. Best performance by an individual country or region does not necessarily lead to improved global air quality in total. To address this situation, this study utilizes a stochastic version of the DEA PO, referred to as SDEA PO, developed in [6].

The SDEA PO does not consider the DMUs in a group as peers having different efficiency levels. Instead, it treats the DMUs as the occurrences of a virtual stochastic DMU. From this perspective, the group of DMUs is a stochastic system, characterized by the joint probability distribution of its inputs and outputs. In this scenario, the efficiency index of the virtual stochastic DMU is a random variable. The mathematical expectation of this random variable is considered as an efficiency measure of the whole group of the DMUs and is referred to as a group efficiency index *E_g_*.

In these settings, each input and output are normalized to the corresponding minimal or maximal values in the group, respectively, as in Formula (3). Next, each-of them is considered as a sample of the occurrences of the independent identically distributed random variables having the Beta distribution. Using this distribution allows for the accommodation of almost all shapes of the empirical data that may be encountered in applications of this method. We consider the group of the DMUs as a sample and use it for the estimation of the parameters of the virtual DMU’s probability distribution. Formula (3) is the basis for the computations. (See [6] for detail.)

As compared with the basic version of the SDEA PO, this study follows publications [7,8] and determines the direction in which the local increase in the group efficiency is the greatest. The goal is achieved when the structural change follows the gradient vector – the direction that is locally optimal. Following the gradient allows for the maximal possible increase in the energy-environmental efficiency at each step that is important for international specialization, trade, and cooperation. (See [9] for details.)

This study utilizes the following SDEA PO model:

(4) $$\begin{array}{l} E_{g}=\frac{u_{\mathrm{GP}}d_{\mathrm{GP}}+u_{\mathrm{RE}}d_{\mathrm{RE}}}{u_{\mathrm{EG}}d_{\mathrm{EG}}+u_{\mathrm{CE}}d_{\mathrm{CE}}},\\ d_{GP},d_{RE},d_{EG,}d_{CE}\;\geq 0,\\ u_{GP},u_{RE},u_{EG,}u_{CE}\;\geq 0, \end{array}$$

where *E_g_* is the mathematical expectation of the group efficiency index, *d_GP_* is the gross domestic product per capita, *d_RE_* is the share of renewable energy in total energy consumption, *d_EG_* stands for energy intensity of the gross output, and *d_CE_*, is the carbon dioxide intensity of energy. The weight coefficients *u_GP_*, *u_RE_*, *u_EG_*, and *u_CE_* are determined as solutions to the linear programming problem. They are the functions of time, and change accordingly, as the DEA inputs and outputs *d_GP_*, *d_RE_*, *d_EG_*, and *d_CE_* change. Publications [[6], [7], [8]] utilize SDEA PO and combine all of the elements of the Formula (4) in one indicator – the group efficiency index. They also present an algorithm and computer program in R language aimed at the estimation of the group efficiency index E_g_ and its gradient. This paper makes a step further by proposing a system of differential-algebraic equations directing the structural change along the gradient at each point in time.

## Differential-algebraic equations of locally optimal trajectory of structural change

This section introduces a system of differential-algebraic equations that governs the process of economic restructuring in the direction of the gradient of the mathematical expectation of the group efficiency index *E_g_*. To derive the equations, we note that the mathematical model (3), (4) defines the group efficiency index *E_g_* as a function of a statistical sample. The data included in the sample comprise the country or a region gross domestic product per capita *d_GP,i_*, and the ratios of the shares of renewable energy to total energy consumption *d_RE,i_*, energy consumption to the gross output *d_EG,i_*, and carbon dioxide emissions per unit of energy consumption *d_CE,i_*. The lower index *i* stands for the ordinal number of a country or region. The omission of this index means the value for the global economy as a whole. In these settings, we compute the gradient of the group efficiency index numerically. The system of differential-algebraic equations devised below directs the structural change alongside the gradient. The obtained trajectory is locally optimal, since it provides a maximum possible increase in the group efficiency index per unit of displacement at each point in time.

Let $\nabla E_{g}$ be the gradient vector of the function *E_g_*, comprising subvectors $\nabla E_{GP}$, $\nabla E_{RE}$, $\nabla E_{EG}$, and $\nabla E_{CE}$:

(5) $$\nabla E_{g}=<\nabla E_{GP},\nabla E_{RE},\nabla E_{EG},\nabla E_{CE}{>}^{T},$$

where upper index *T* stands for the transposition, and lower indexes *GP*, *RE*, *EG*, and *CE* stand for the gross domestic product per capita, shares of renewable energy in total energy consumption, energy intensity of the gross output, and carbon dioxide intensity of energy *d_CE,i_*, respectively.

The system of differential-algebraic equations governing the movement alongside the gradient is as follows:

(6) $$\begin{array}{l} \frac{d}{dt}d_{GP}=\gamma_{\mathrm{GP}}(t)\nabla E_{GP},\\ \frac{d}{dt}d_{RE}=\gamma_{\mathrm{RE}}(t)\nabla E_{RE},\\ \frac{d}{dt}d_{EG}=\gamma_{\mathrm{EG}}(t)\nabla E_{EG},\\ \frac{d}{dt}d_{CE}=\gamma_{\mathrm{CE}}(t)\nabla E_{CE}, \end{array}$$

where ***d****_GP_*, ***d****_RE_*, ***d****_EG_*, and ***d****_CE_* are the vectors with the components *d_GP_,_i_*, *d_RE,i_*, *d_EG,i_*, and *d_CE,i_*, correspondingly. Matrix-valued functions ***γ****_GP_()*, ***γ****_RE_()*, ***γ****_EG_()*, and ***γ****_CE_()* determine the rate of structural change in a particular collection of the relative indicators country- or region-wise, respectively. They are the diagonal matrices with the elements corresponding to the desired rate of change of the corresponding indicator in a given country or region. Their values may be estimated as magnitudes of the observed structural changes, correspondingly. These matrix-valued functions are exogenous variables in this model. Each equation in a group in Formula (6) represents a system of differential equations, each relating to a specific indicator, and inside the group, the differential equations pertain to a particular country or region. The total number of equations in Formulas (6) is four times the number of the DMUs in the group.

The components of the vectors ***d****_GP_*, ***d****_RE_*, ***d****_EG_*, and ***d****_CE_*, are subject to the non-negativity and boundary constraints:

(7) $$\begin{array}{l} 0<a_{GP}\leq d_{GP}\;\leq b_{GP},\\ 0<a_{RE}\leq d_{RE\;}\leq \;b_{RE},\\ 0<a_{EG}\leq d_{EG}\leq b_{EG},\\ 0<a_{CE}\leq d_{CE}\leq b_{CE}, \end{array}$$

where the components of the vectors ***a****_GP_*, ***a****_RE_*, ***a****_EG_*, ***a****_CE_*, ***b****_GP_*, ***b****_RE_*, ***b****_EG_*, and ***b****_CE_* are economically justified lower and upper boundaries, respectively.

The shares of the quantitative indicators in total and the variables included in the system of Eq. (6) are related as follows:

(8) $$\begin{array}{l} d_{GP,i}=d_{G,i}/d_{P,i},\\ d_{RE,i}=d_{R,i}/d_{E,i},\\ d_{EG,i}=d_{E,i}/d_{G,i},\\ d_{CE,i}=d_{C,i}/d_{E,i}, \end{array}$$

where *d_G,i_*, *d_P,i_*, *d_R,i_*, *d_C,i_*, and *d_E,i_* are the shares of the country or region in total for the gross domestic product, population, renewable energy, carbon dioxide emissions, and energy consumption, correspondingly. The shares should satisfy the boundary and summation-to-one conditions:

(9) $$\begin{array}{l} 0<a_{G,i}\leq d_{G,i}\leq b_{G,i}\leq 1,\\ 0<a_{P,i}\leq d_{P,i}\leq b_{P,i}\leq 1,\\ 0<a_{R,i}\leq d_{R,i}\leq b_{R,i}\leq 1,\\ 0<a_{C,i}\leq d_{C,i}\leq b_{C,i}\leq 1,\\ 0<a_{E,i}\leq d_{E,i}\leq b_{E,i}\leq 1, \end{array}$$

and

(10) $$\begin{array}{l} \sum d_{G,i}=1,\\ \sum d_{P,i}=1,\\ \sum d_{R,i}=1,\\ \sum d_{C,i}=1,\\ \sum d_{E,i}=1,\; \end{array}$$

where *a_G,i_*, *b_G,i_*, *a_P,i_*, *b_P,i_*, *a_R,i_*,*b_R,i_*, *a_C,i_*, *b_C,i_*, *a_E,i_*, and *b_E,i_* are economically justified lower and upper boundaries of the shares of countries or regions in gross domestic product, population, renewable energy, CO_2_ emissions, and energy consumption, respectively. The summation in Formula (10) is done by lower index *i* – an ordinal number of a country or region in the group. By adding non-negative slacks, the systems of inequalities (7) and (9) may be rewritten as the systems of algebraic equations, satisfying non-negativity constraints.

By doing so, we arrive at the system of differential – algebraic equations (SDAE) (6), (7), (8), (9), (10) with non-negativity constraints imposed on all variables *d_G,i_*, *d_P,i_*, *d_R,i_*, *d_E,i_*, *d_GP,i_*, *d_RE,i_*, *d_EG,i_*, and *d_CE,i_*, and the slacks used to transform inequalities (7) and (9) into equalities.

When setting up the lower and upper boundaries in Formulas (7), (8), (9), the boundary values in Formulas (7) and (9) should satisfy the following conditions:

(11) $$\begin{array}{l} a_{GP,i}\;\geq a_{G,i}/b_{P,i},\\ b_{GP,i}\leq b_{G,i}/a_{P,i},\\ a_{RE,i}\;\geq a_{R,i}/b_{E,i},\\ b_{RE,i}\leq b_{R,i}/a_{E,i},\\ a_{EG,i}\;\geq a_{E,i}/b_{G,i},\;\\ b_{EG,i}\leq b_{E,i}/a_{G,i},\\ a_{CE,i}\;\geq a_{C,i}/b_{E,i},\;\\ b_{CE,i}\leq b_{C,i}/a_{E,i}. \end{array}$$

To solve the system of Eqs. (6), (7), (8), (9), (10), initial conditions should be set up, and one group of the structural indicators: *d_G,i_*, *d_P,i_*, *d_R,i_*, *d_C,i_*, or *d_E,i_* -- the shares of the country or region in total, should be considered as exogenous variables. We suggest in this study that this role is assigned to the indicator of population:

(12) $$d_{P,i}=p_{i}(t),\;$$

where *p_i_()* is a known non-negative continuous function of time *t*, satisfying the corresponding parts of the conditions (9) and (10); where lower index *i* stands for a country or region.

Formulas (5), (6), (7), (8), (9), (10), (11), (12) define a system of differential – algebraic equations (SDAE) with non-negativity constraints, describing the trajectory of locally optimal environmental friendly structural change in the global economy. Theory, practice of applications, and R programs for solution of SDAEs are provided in [10] and earlier publications in this series, and publications [11] and [12]. Algorithms aimed at the inclusion of the non-negativity constraints are considered in [13].

Practical applications of the suggested model depend on the availability of the corresponding computer software. R-language and computer algebra systems Maple, Matlab, and Mathematica have the means for solving SDAEs numerically. However, to the best of the author’s knowledge, only Matlab 2019 allows for the non-negativity constraints in the SDAEs. The software expansion required by the proposed model may stimulate further development of the computer algebra systems and result in a variety of applications of the suggested model.
